# Successful treatment of a fracture of a huge Achilles tendon ossification with autologous hamstring tendon graft and gastrocnemius fascia flap: a case report

**DOI:** 10.1186/s12891-015-0821-x

**Published:** 2015-11-24

**Authors:** Hisatoshi Ishikura, Naoshi Fukui, Hiroshi Takamure, Satoru Ohashi, Mitsuyasu Iwasawa, Kentaro Takagi, Ayako Horita, Ikuo Saito, Toshihito Mori

**Affiliations:** Department of Orthopaedic Surgery, National Hospital Organization, Sagamihara Hospital, 18-1 Sakuradai, Minami-ku, Sagamihara City, Kanagawa Prefecture 252-0314 Japan; Department of Pathology, National Hospital Organization, Sagamihara Hospital, 18-1 Sakuradai, Minami-ku, Sagamihara City, Kanagawa Prefecture 252-0314 Japan

**Keywords:** Achilles tendon, Ossification, Fracture, Reconstruction, Hamstring tendon

## Abstract

**Background:**

Fracture of an ossified Achilles tendon is a rare entity, and no standard treatment has been established. This is the first report to describe the use of a hamstring tendon graft and gastrocnemius fascia flap for Achilles tendon reconstruction.

**Case presentation:**

We present the case of a 50-year-old woman with fracture of an ossified Achilles tendon. She presented to our clinic with acute right hindfoot pain, which started suddenly while going up the stairs. Plain radiography and magnetic resonance imaging revealed a massive ossification on the right Achilles tendon extending over 14 cm in length; the ossification was fractured at 5 cm proximal to the calcaneus insertion. Surgical treatment included removal of the ossified tendon and reconstruction with an autologous hamstring tendon graft and gastrocnemius fascia flap. One year after surgery, she was able to walk with little pain or discomfort and to stand on her right tiptoe.

**Conclusion:**

Our novel surgical procedure may be useful in the treatment of fractured ossified Achilles tendons and large Achilles tendon defects.

## Background

Ossification of an Achilles tendon is a rare entity. Furthermore, only a few cases of fracture of an ossified Achilles tendon (FOAT) have been reported [1–7]. In previous reports, FOAT was treated either conservatively or surgically by excision and reconstruction using gastrocnemius (GC) fascia flaps, tensor fascia latae grafts, or adjacent tendons. However, to date, no standard treatment methods have been established for FOAT. In addition, currently available methods may not be effective in treating extensive ossifications, which would leave large defects after excision. We report the case of a fracture of a huge Achilles tendon ossification that was successfully treated by ossification removal and tendon reconstruction with an autologous hamstring tendon graft and GC fascia flap. Our novel surgical procedure for FOAT showed promising short-term results. To the best of our knowledge, this is the first report to describe the use of a hamstring tendon graft for reconstruction of FOAT.

## Case presentation

### Case report

**Fig. 1:**
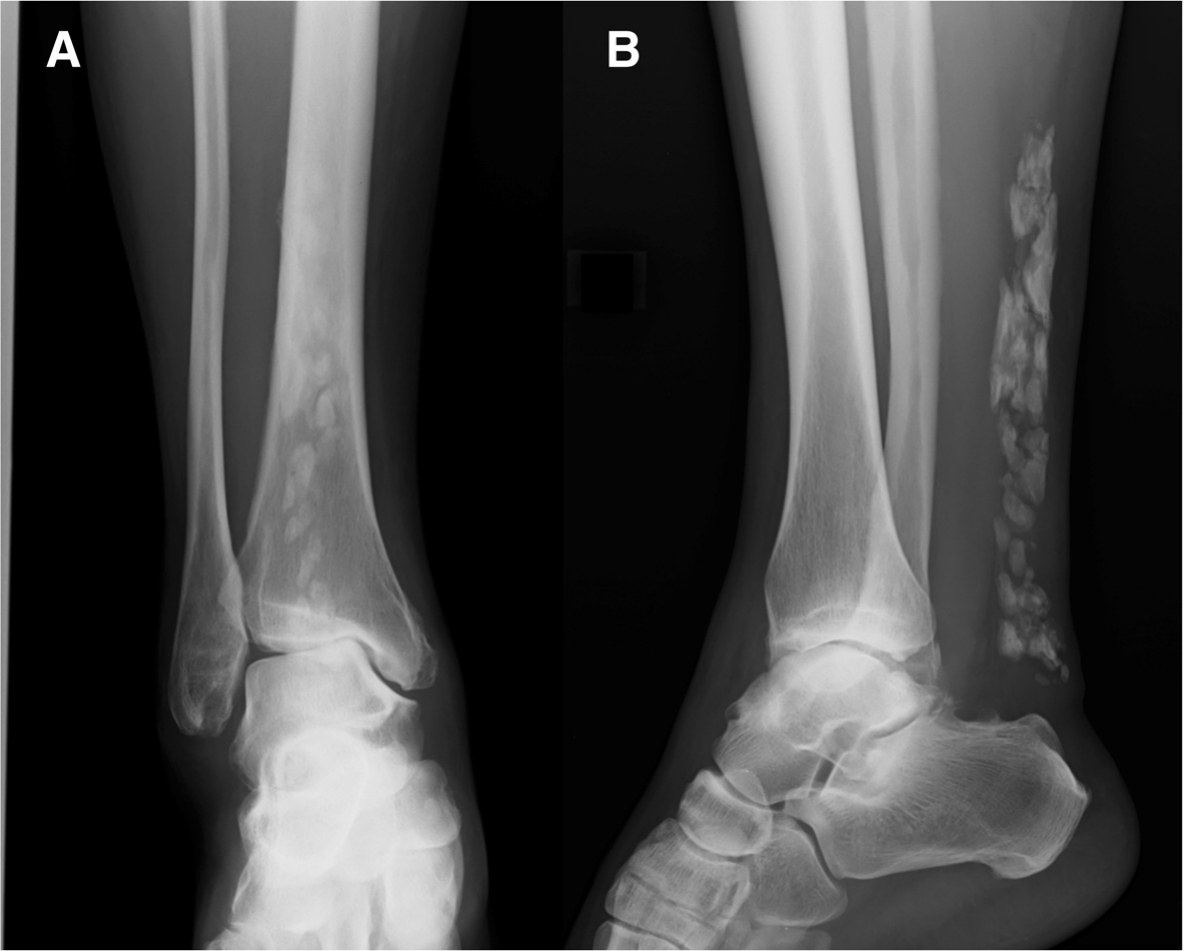
Plain radiographs taken at the patient’s first visit. Anteroposterior (**a**) and lateral radiographs (**b**) of the right ankle showed extensive ossification of the Achilles tendon and osteoarthritis of the ankle joint

**Fig. 2:**
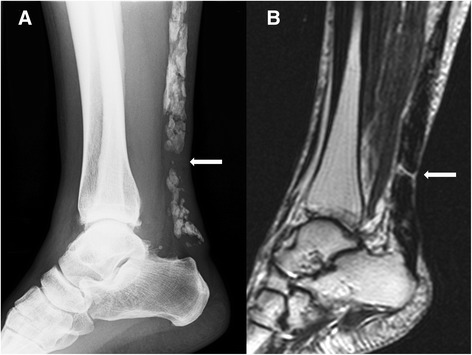
Radiographic and magnetic resonance images of the Achilles tendon before surgery. Lateral view radiograph (**a**) revealed a gap within the ossified mass (arrow). Sagittal proton density and T2-weighted magnetic resonance images (**b**) showed a clear high-signal intensity line across the distal one-third of the tendon, indicating a fracture in the ossified lesion (arrow). No edema or effusion was observed around the fracture site

A 50-year-old woman (height 155 cm, weight 54 kg) with right hindfoot pain was referred to our clinic in April 2014. She had experienced discomfort in her right hindfoot over the last several years. The discomfort had gradually worsened over the past several months. She was working as a full-time bank employee, and hindfoot pain on standing and walking was making her train commute to work difficult. She had no significant medical, family, or psychosocial history. Ankle range of motion (ROM) was limited to 10° for dorsal flexion and 40° for plantar flexion. Physical examination revealed diffuse ankle swelling, Achilles tendon stiffness, and moderate tenderness to palpation. Despite this discomfort, she could walk stably without a limp and stand on her tiptoes. Radiographs revealed extensive Achilles tendon ossification and osteoarthritis of the ankle joint (Fig. 1a, b). The length of the ossification was approximately 14 cm. She was prescribed oral anti-inflammatory agents. Ten days later, she felt a sudden pain in her right hindfoot while going up the stairs. The pain progressively worsened, causing her to limp. She visited our clinic several days after the incident. Ankle ROM was reduced to −5° for dorsal flexion and 30° for plantar flexion. Palpation revealed a severely tender defect 5 cm proximal to the Achilles tendon insertion. She could not stand on her right tiptoe and had a positive Thompson test. A radiograph of her right Achilles tendon showed a gap within the ossified mass, indicating FOAT (Fig. 2a). Sagittal proton density and T2-weighted magnetic resonance imaging demonstrated a high signal intensity line at the fracture site (Fig. 2b). The MRI also indicated that her gastrocnemius and soleus muscles were rather well preserved. Blood and radiological tests were performed to rule out the presence of systemic disease, and no abnormalities were detected.

**Fig. 3:**
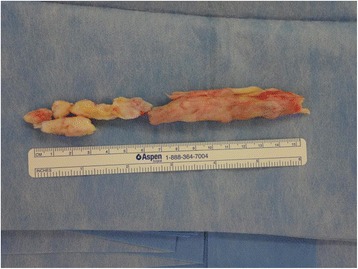
Photograph of the excised tendon. The ossified tendon was removed in several pieces. The largest piece was 9.3 cm in length

Surgery was performed with the patient in the prone position. A longitudinal midline skin incision was made directly above the Achilles tendon to expose the bony mass. The fracture site was 5 cm proximal to the tendon insertion. The proximal part of the ossified tendon, which was 9.3 cm in length, was excised en bloc. The distal part was removed with several blocks (Fig. 3). The defect was 12 cm in length, even with the ankle in maximal plantar flexion.

**Fig. 4:**
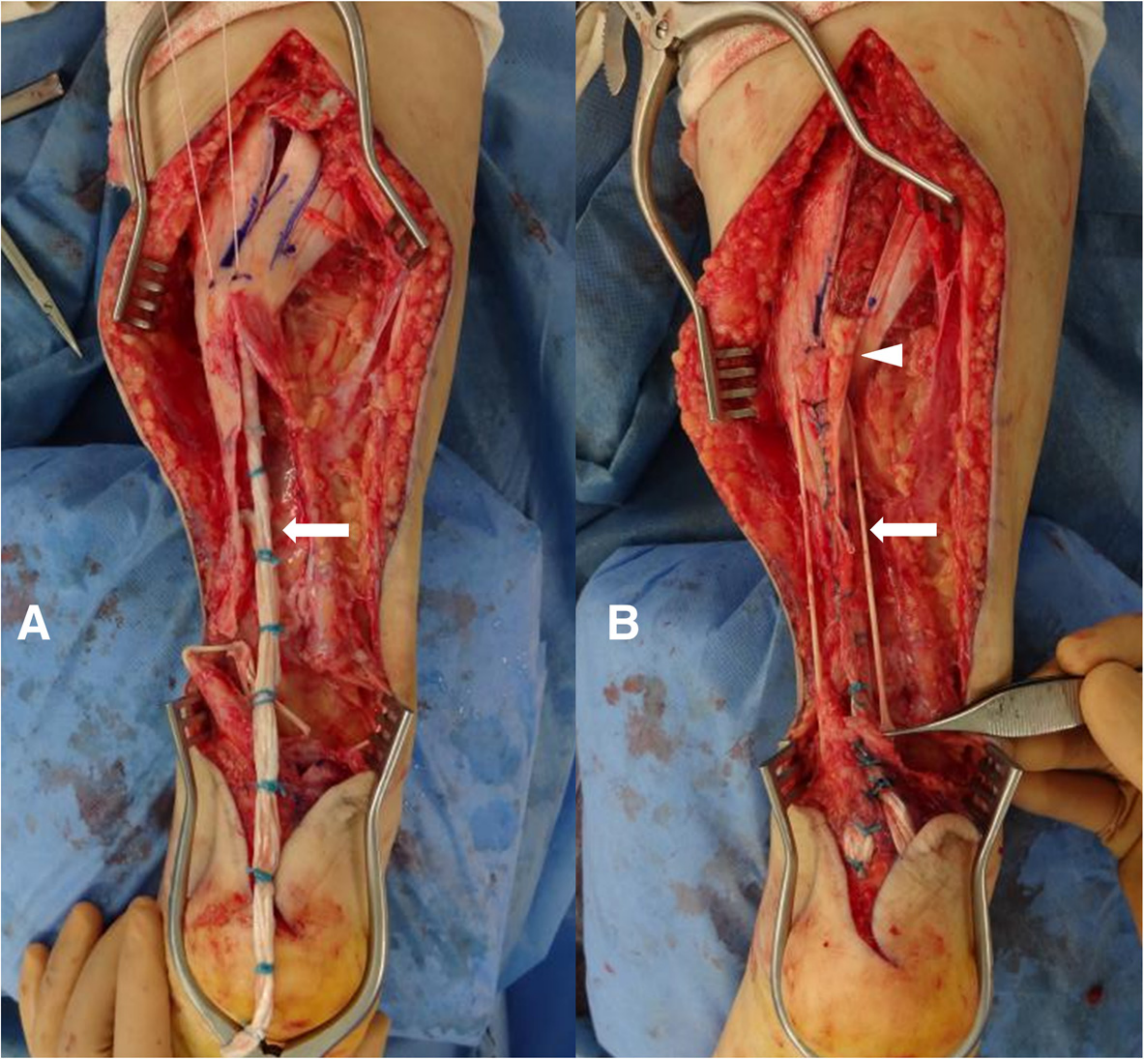
Intraoperative view of the reconstructed tendon. The proximal parts of the hamstring tendon grafts (*arrow*) were sutured together, passed through the GC fascia, and fixed to the proximal Achilles tendon stump (**a**). The tendon graft was augmented with a GC fascia flap (arrowhead) and plantaris tendon (*arrow*) (**b**). GC, gastrocnemius

After ossification removal, the hamstring tendons were obtained for reconstruction of the Achilles tendon. A 4-cm oblique incision was made over the pes anserinus, and the ipsilateral semitendinosus and gracilis tendons were harvested proximally using an open-ended tendon stripper while the knee was held at 30° of flexion to reduce the tension on the tendons. The tendons were then freed distally at the tibial insertion. Both tendons were approximately 20 cm long. After removing muscle tissues attached to the tendons, the tendons were bundled together, and the proximal part of the hamstring tendon graft was tied using a baseball suture technique with 1–0 threads. The ends of these threads were passed through the GC fascia and then sutured to the proximal Achilles tendon stump (Fig. 4a). The distal part of the graft was passed through a small incision in the substance of the distal Achilles tendon stump in a medial to lateral direction and then overturned proximally. With the ankle in maximal plantar flexion, the graft was sutured to the stump at the entry and exit points. As the plantaris tendon remained intact, the proximal part of this tendon was harvested with a tendon stripper and folded in half to reinforce the reconstruction. Finally, the proximal part of the graft was reinforced by turning the GC fascia flap downwards (Fig. 4b).

Postoperatively, the involved ankle was immobilized for 4 weeks in a below-knee non-weight-bearing cast with approximately 20° of plantar flexion. After removing the cast, she was instructed to wear a hinged ankle-foot orthosis that permitted full plantar flexion but limited dorsiflexion to a set angle [8], and to perform active ROM exercises. At week 7, full weight-bearing with the orthosis was permitted, and passive dorsiflexion exercises were initiated. At week 10, the orthosis was removed, and ROM exercises and gait training were continued. ROM exercises and gait training were performed under the supervision of the same experienced physical therapist. No adverse events occurred during the postoperative period.

One year after the operation, she had little pain and discomfort in the right Achilles region and was able to stand on her right tiptoe. Ankle ROM for dorsiflexion and plantar flexion was 15° and 45°, respectively, which was almost the same as that of the contralateral side. The American Orthopedic Foot and Ankle Society (AOFAS) Ankle/Hindfoot Scale score [9] and Achilles tendon total rupture score (ATRS) were used to evaluate preoperative and postoperative clinical status [10]. One year after surgery, the AOFAS score and ATRS increased from 50 to 75 and 23 to 56, respectively. Further improvements in these scores were limited by anterior ankle pain that remained after surgery, which was attributed to osteoarthritis of the ankle joint.

**Fig. 5:**
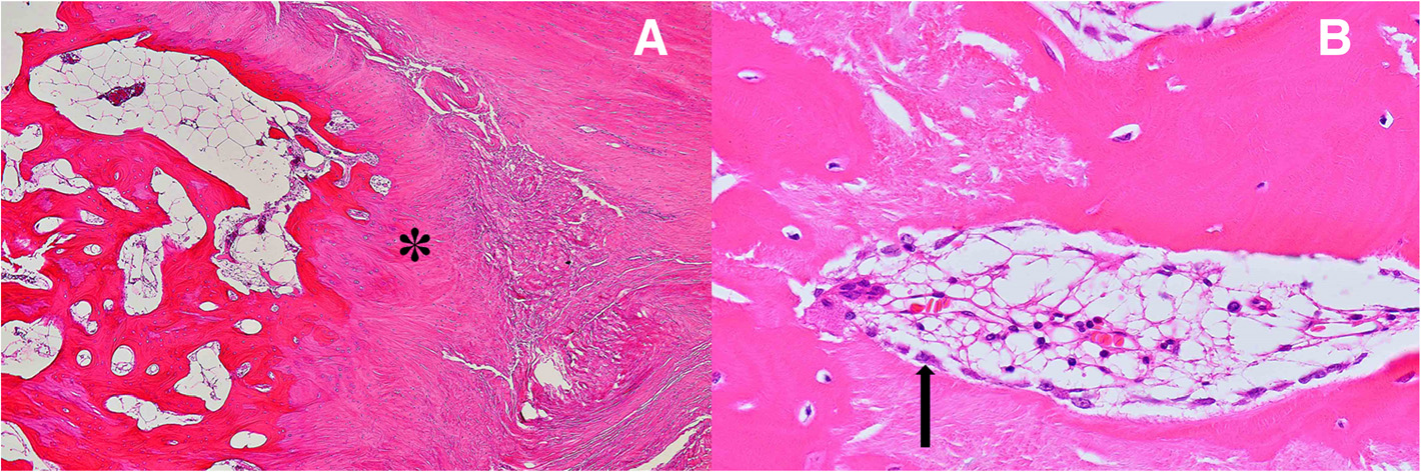
Hematoxylin and eosin staining of the ossified tendon. Collagenous tendon tissue underwent cartilaginous metaplasia (✽) and was gradually replaced by lamellar bone with fatty bone marrow (**a**). In some areas, the surface of the lamellar bone was covered by a number of osteoblasts (*arrow*), suggesting vigorous osteoblastic activity (**b**). Magnification, ×40 (**a**) and × 400 (**b**)

Histologically, the ossified tendon showed a mixture of fibrous tissue, cartilaginous tissue, and lamellar bone with little inflammatory cell infiltration. These findings indicated that part of the collagenous fiber of the tendon underwent cartilaginous metaplasia and was gradually replaced by lamellar bone (Fig. 5a, b). The lamellar bones were surrounded by many osteoblasts, suggesting endochondral ossification of the Achilles tendon.

### Discussion

Achilles tendon ossifications are rare and usually asymptomatic, particularly those that are small and localized. Although the exact mechanism of ossification is unknown, the most common proposed etiologies are previous surgery and trauma [1–3, 11–15]. Infectious, metabolic, and systemic diseases such as syphilis, GC abscess, gout, diabetes, Wilson’s disease, ochronosis, diffuse idiopathic skeletal hyperostosis, Reiter’s syndrome, and ankylosing spondylitis may also cause ossification [1–4, 11, 14]. In our case, none of these diseases was present, and the laboratory data were unremarkable. The only cause applicable to our patient was previous trauma. The patient had sustained a right ankle dislocation, which was repositioned manually, and a partial rupture of the right Achilles tendon while playing high school basketball (Table 1). She did not undergo surgery or improper conservative treatment (e.g., prolonged cast immobilization) for these injuries. Therefore, the Achilles tendon ossification and osteoarthritis of the ankle joint were likely due to injury-related trauma and not treatment-related trauma.

**Table 1:** Timeline

1980	Dislocation of the right ankle Partial rupture of the right hindfoot
2008	Discomfort in the right hindfoot
2014.4	Achilles tendon ossification
	Osteoarthritis of the ankle joint
2014.4	Oral anti-inflammatory drugs
2014.4	Fracture of ossified Achilles tendon
2014.5	Ossification removal and reconstruction using autogous hamstring graft and gastrocnemius fascia flap
2015.5	Little pain or discomfort in the right Achilles region
	Mild anterior ankle pain due to osteoarthritis of the ankle joint

Only a few cases of FOAT have been previously reported. No standard treatments for FOAT have been established, and the optimal treatment for this condition remains a matter of debate; however, both conservative and surgical treatments have been proposed. Goyal et al. reported that conservative treatment of FOAT produced a satisfactory functional result in an 84-year-old man [3]. However, given the younger age and higher activity level of our patient, a conservative approach was not likely to produce the desired functional outcome. Resnik et al. reported a case of 36-year-old man who underwent conservative treatment for FOAT. The patient experienced persistent pain and swelling and eventually underwent surgical excision of the bony mass and tendon reconstruction [5]. Several other reports have also recommended surgical treatment of FOAT [1, 6, 13], including internal fixation of the fractured mass [16] and surgical excision and reconstruction using GC fascia flaps [4, 11, 17] or tensor fascia latae grafts [5]. FOAT may also be treated by an adjacent tendon transfer; the use of the flexor hallucis longus tendon and a peroneus brevis tendon has been reported [7, 18].

The current case is unique in the longer length of the ossification compared with previously reported cases. Thus, previous reconstruction methods would not have been sufficient to repair the large gap remaining after excision of the mass. Meanwhile, fixation of the fractured mass may not be the treatment of choice because of the risks of fracture non-union and refracture. To determine the appropriate tissues to use for the reconstruction, we referred to previous reports of chronic Achilles tendon ruptures. Semitendinosus and gracilis tendons have both been used for the reconstruction of large defects caused by chronic Achilles tendon ruptures and have yielded good clinical and functional results [19–22]. Considering this and the patient’s activity level, we initially planned to reconstruct the Achilles tendon using the double-folded semitendinosus and gracilis tendons. Although good results have been reported with tendon transfer techniques [7, 18], we did not consider those methods because connecting the proximal and distal stump firmly using a free tendon graft may better restore continuity and muscle function and strength compared to tendon transfer. We propose that free tendon grafting is a more suitable surgery to address the large gap in FOAT; however, the method may have several drawbacks, such as the need for another incision and the complexity of the surgical procedure, which may thus increase the risk of complications.

During surgery, we found that the defect in the tendon was too large to be reconstructed by double-folded tendons. Thus, the hamstring tendons were grafted as single bands, and a GC fascia flap and a plantaris tendon were added to augment it [19, 23]. The use of multiple tissues provided sufficient strength to the reconstructed tendon. This is the primary advantage of our procedure, which likely contributed to the satisfactory result in our patient. Since a chronically disrupted Achilles tendon has been successfully reconstructed with a semitendinosus or gracilis tendon alone [19–23], there may be concern that the reconstructed tendon might have been over-augmented in our procedure. Admitting this possibility, we still believe that our method is a viable option for Achilles tendon reconstruction, especially when the gap is extremely large and the patient is young and active.

In the present patient, the distal part of the Achilles tendon was not ossified, which allowed us to stably fix the graft to the tendon stump distally. However, if the distal end of the Achilles tendon was affected and required excision, it would be an issue as to how the graft was to be fixed to the calcaneus. A recent study has shown that the calcaneal insertion may be successfully reconstructed with the use of an interferential screw [22].

In the present case, the ossified mass did not exhibit a unique histology despite its exceptional size. Previous studies have shown that Achilles tendon ossifications exhibit a variety of histological findings, including endochondral and intramembranous ossification, lamellar bone, and conglomerate foci of calcification [24]. However, to date, cartilaginous metaplasia of fibroblasts and subsequent endochondral ossification is the most accepted hypothesis for the pathogenesis of Achilles tendon ossifications [11]. The histological findings in our case are consistent with this hypothesis. Therefore, the large size of the ossification in our case may not be related to the etiological cause. We speculate that the lack of treatment of the initial traumatic injuries may be responsible for the extensive ossification.

## Conclusion

We describe the case of a fracture of an extensive Achilles tendon ossification that was successfully repaired using a novel tendon reconstruction method consisting of a combination of autologous hamstring tendon graft and GC fascia flap. This reconstruction method may be used for treating not only FOAT but also large Achilles tendon defects due to trauma, malignancy, or other causes.

## Consent

Written informed consent was obtained from the patient for publication of this case report and any accompanying images. A copy of the written consent is available for review by the Editor of this journal.

## Abbreviations

AOFAS: American Orthopedic Foot and Ankle Society
ATRS: Achilles tendon total rupture score
FOAT: fracture of ossified Achilles tendon
GC: gastrocnemius
ROM: range of motion
